# Melatonin prevents morphine-induced hyperalgesia and tolerance in rats: role of protein kinase C and N-methyl-D-aspartate receptors

**DOI:** 10.1186/1471-2253-15-12

**Published:** 2015-01-28

**Authors:** Li Song, Chaoran Wu, Yunxia Zuo

**Affiliations:** Translational Medical Neuroscience Center, West China Hospital Sichuan University, Chengdu, Sichuan 610041 China; Department of Anesthesiology, West China Hospital Sichuan University, Chengdu, Sichuan 610041 China

**Keywords:** Melatonin, Morphine-induced hyperalgesia, Morphine tolerance, PKCγ, NR1

## Abstract

**Background:**

Morphine-induced hyperalgesia and tolerance significantly limits its clinical use in relieving acute and chronic pain. Melatonin, a pineal gland neurohormone, has been shown to participate in certain neuropsychopharmacological actions. The present study investigated the effect of melatonin on morphine-induced hyperalgesia and tolerance and possible involvement of protein kinase C (PKC)/N-methyl-D-aspartate (NMDA) pathway in melatonin-mediated.

**Methods:**

Experiments were performed on adult, male Sprague–Dawley rats. Melatonin (10 mg/kg, intraperitoneal, i.p.) or saline was administrated 10 min after morphine injection (10 mg/kg, subcutaneous, s.c.) each day for consecutive 14 days. Withdrawal threshold of the hindpaw to mechanical and thermal stimulation was measured before any drug administration and one hour after melatonin or saline on each designated test day. On the 15^th^ day, thermal withdrawal was measured after s.c. morphine (20 mg/kg), but not melatonin, and morphine tolerance was measured and expressed by MPAE% (percent of maximal possible anti-nociceptive effect) of morphine. Levels of expression of protein kinase C gamma (PKCγ) and NMDA receptor subtype NR1 in spinal cord were detected by Western blotting.

**Results:**

The mechanical withdrawal threshold and thermal withdrawal latency decreased and shortened significantly (i.e., threshold decreased) in rats that received morphine treatment for two weeks compared with that in rats receiving saline. This morphine-induced mechanical and thermal hyperalgesia were greatly attenuated by co-administration of morphine with melatonin. The MPAE% representing morphine analgesic effect was reduced approximately 60% in rats that received morphine treatment. However, following the treatment of morphine with melatonin, the MPAE% was reduced only about 30%, comparing with those that received saline treatment as control. Administration of morphine alone resulted in significantly increased expression of PKCγ and NR1 proteins in the spinal cord. These increased levels of expression of PKCγ and NR1 were significantly inhibited by co-administration of morphine with melatonin.

**Conclusions:**

Our findings demonstrate that melatonin have potential to attenuate repetitive morphine-induced hyperalgesia and tolerance, possibly by inhibiting PKCγ and NR1 activities in the spinal cord.

## Background

Opioids such as morphine are effective analgesics that are widely used in relieving acute and chronic pain [1]. However, repeatitive administration of morphine may cause hyperalgesia [2, 3], in addition to inducing analgesic tolerance [4–7], a diminished morphine analgesic effect. Morphine-induced hyperalgesia and analgesic tolerance exhibit similar clinical manifestations, e.g., to reach an adequate analgesic effect, it is necessary to increase doses of morphine over time [3]. Therefore, morphine-induced hyperalgesia and tolerance become a real challenge that limits a lot of the clinical use of morphine. In the past 20 years, many studies discussing mechanisms of morphine-induced hyperalgesia and tolerance have been focusing on changes of neuronal plasticity in the central nervous system (CNS) [6]. Such neuronal plastic changes involve activation of excitatory amino acid receptors, the subsequent intracellular cascades including translocation and activation of PKC as well as nitric oxide production, leading to the functional modulation of receptor-ion channel complexes (e.g. the desensitization of μ-opioid receptor) [4, 5, 7–16]. Studies have shown that activations of PKC and NMDA receptor in the spinal cord play critical roles in the development of morphine-induced hyperalgesia and tolerance [5, 7, 10, 15, 17]. Intrathecal co-administration of morphine with GM1 ganglioside (a intracellular inhibitor of PKC translocation) or chelerythrine chloride (a non-specific PKC inhibitor) effectively attenuates the development of the tolerance to morphine’s analgesic effect in rats [4, 10, 15]. Likewise, intrathecal co-administration of morphine with MK-801 (a non-competitive NMDA receptor antagonist) or LY274614 (a competitive NMDA receptor antagonist) also effectively prevents the development of morphine tolerance in animals with or without nerve injury [17, 18]. Thus, inhibition of PKC and/or NMDA receptor activities in the spinal cord may be able to effectively prevent the morphine-induced hyperalgesia and tolerance.

Melatonin (*N*-acetyl-5-methoxytryptamine), a pineal gland neurohormone synthesized from L-tryptophan, has been demonstrated to play an important role in the biologic regulation of circadian rhythms, sleep, mood, reproduction, tumor growth, and neuroprotection [19–21]. Recent evidences have shown that systemic or intrathecal administration of melatonin results in dose-dependent analgesic effect and prevents morphine-induced hyperalgesia [3, 22–25]. However, the cellular mechanisms underlying such interaction of melatonin and morphine remain elusive.

The present study was first to examine and confirm the possible inhibitory effect of co-administration of melatonin with morphine on morphine-induced hyperalgesia and tolerance, then further to investigate the possible role of PKCγ and NR1 activities in the spinal cord in melatonin-induced reduction of morphine-induced hyperalgesia and tolerance.

## Methods

This study was conducted with the approval of the Institutional Animal Experimental Ethics Committee of Sichuan University (Chengdu, China). The experimental protocol was approved by Institutional Animal Care and Use committee of Sichuan University.

### Experimental animals

Adult male Sprague–Dawley rats weighing 200 ± 50 g (n = 24) provided by Sichuan University Medical Animal Center (Chengdu, China) were used in this study. Rats were housed in individual cages with free access to water and food. Room temperature was maintained at 24°C with a 12-hour light/darkness cycle. All experiments were conducted during the period of 9 am to 4 pm on each test day.

### Drugs and treatments

Morphine hydrochloride (Shenyang First Pharmaceutical Factory, Shen Yang, China) and melatonin (Sigma, St. Louis, MO, US) were dissolved in saline.

Rats were randomly assigned to receive subcutaneous (s.c.) administration of saline (1 ml/kg) or morphine (10 mg/kg) or intraperitoneal (i.p.) saline or melatonin (10 mg/kg). Melatonin or saline was administrated 10 min after morphine injection. Each regimen was given once daily for consecutive 14 days. Based on our previous study, repetitive morphine treatment (10 mg/kg, s.c) given once daily for 14 days can produce the tolerance to morphine’s analgesic effect [15]. Moreover, according to Raghavendra’s study, co-administration with morphine, melatonin in dose of 10 mg/kg can significantly reverse the morphine tolerance and dependent [26]. To avoid the acute effect of melatonin on nociceptive response, on day 15 (24 h after the last treatment of consecutive 14 days co-administration), all rats were injected with morphine (20 mg/kg, s.c.) alone to further assess the tolerance to morphine’s analgesic effect.

### Behavioral assessments

Animals were habituated to the testing environment for 1 h each day for consecutive 3 days before the first behavioral test. Habituation consisted of moving rats from their home room to the testing room and keeping them in the testing apparatus for 1 h. The behavioral experimenters were blinded to the drug administration. Mechanical withdrawal threshold and thermal withdrawal latency of all rats were measured on day 0 (before drug administration), 1, 3, 5, 7 and 14. To avoid the acute effect of melatonin, morphine or the combination of morphine and melatonin, on nociceptive response, baseline nociceptive thresholds (mechanical withdrawal threshold and thermal withdrawal latency) on both of the hindpaws were determined before any drug administration on each designated test day. The morphine tolerance was measured at 60 min after co-administration (10 mg/kg melatonin and/or 10 mg/kg morphine) from day 1 to 14, as well as at 60 min after administration of morphine (20 mg/kg) alone on day 15. Rats were euthanized after the final behavioral test and spinal cord samples were harvested.

#### Mechanical allodynia

Mechanical withdrawal threshold was measured using a von Frey filament set with a calibrated range of bending force (0.6, 1, 1.4, 2, 4, 6, 8, 10, 15, and 26 g) [27]. Each rat was placed into a plastic cage with a wire mesh bottom. A single filament was applied perpendicularly to the plantar surface of the each hindpaw for five times with an interval of 5 s. A positive response was defined as at least one clear withdrawal response out of five applications. Filaments were applied in an up-and-down order according to a negative or positive response to determine the hindpaw withdrawal threshold.

#### Thermal hyperalgesia

Thermal withdrawal latency to radiant heat was determined according to a previously described method using a 390 Analgesia Meter (IITC Inc., Woodland Hills, CA) [28]. Rats were placed individually into plexiglas cubicles placed on a transparent glass surface. The light beam from a projection bulb, located below the glass, was directed at the plantar surface of each hindpaw. Hindpaw withdrawal latency was defined as the time from the onset of radiant heat stimulation to withdrawal of the hindpaw. Radiant heat intensity was adjusted to result in a baseline latency of about 12 s and a cut-off time of 20 s. Three trials with an interval of 5 min were made for each hindpaw and scores from three trials were averaged to yield mean withdrawal latency for each hindpaw.

#### Morphine tolerance

Development of the tolerance to morphine’s analgesic effect was assessed by hindpaw thermal withdrawal latency test [14, 15]. The hindpaw thermal withdrawal latency was converted to MPAE%. MPAE% was determined by comparing the hindpaw withdrawal latency before (basal latency) and after administration (test latency) using the equation: MPAE% = [(test latency - basal latency)/(20–basal latency) × 100% (20 s as the cut-off time). A higher MPAE% represented a better analgesic effect.

### Western blot

Rats in each experimental group (n = 3/6) were deeply anesthetized with pentobarbital (80 mg/kg, i.p.) and decapitated for rapid tissue harvesting. The L4-L5 segment of the spinal cord was first divided into the right and left side and then further separated into the dorsal and ventral horn, respectively. The tissues were dissected and rapidly frozen on dry ice and stored at -80°C for later Western blot analysis. Frozen tissue samples were homogenized in a homogenization buffer (59 mM Tris–HCl, 0.1mM EDTA, 0.1 mM EGTA, 1mM phenymethylsulfonyl fluoride, 1 μM leupeptin, 2 μM pepstain A). The homogenate was centrifuged at 4°C for 10 min at 8,000 × g. The protein concentration of the supernatants was assayed by using a microplate reader (Bio-TeK Instrument Inc. Winooski, VT). Supernatants (50 μg) were heated for 10 min at 100°C and loaded onto 4% stacking/10% separating SDS-polyacrylamide gels for the protein separation. The protein was then electrophoretically transferred onto polyvinylidenedifluoride membrane (Millipore). The membranes were then blocked with 5% non-fat dry milk solution for 1 h and incubated overnight at 4°C with a primary antibody (mouse anti-rabbit PKCγ, 1:100; 84 kDa or rabbit anti-mouse NR1, 1:500; 100 kDa) with moderate shaking. A corresponding horseradish peroxidase -conjugated secondary antibody (Donkey anti-rabbit or mouse, 1:5,000; Amersham Biosciences, Arlington Heights, IL) and chemiluminescent solution (NEN) were used to visualize a blot, followed by exposing the blot onto hyperfilm (Amersham) for 5 min. Blots were then incubated in a stripping buffer (67.5 mM Tris, pH 6.8, 2% SDS, and 0.7% b-mercaptoethanol) for 1 h at room temperature and reprobed with a polyclonal rabbit anti-β-actin antibody (1:10,000; Alpha Diagnostic International, San Antonio, TX) as a loading control. Tissue samples from experimental rats were probed in triplicate. The density of each band was assayed with Adobe Photoshop 7.0 (Adobe Inc.) and normalized against each corresponding β-actin loading control.

### Immunohistochemistry

Rats in each experimental group (n = 3/6) were deeply anesthetized with pentobarbital (80 mg/kg, i.p.) 24 h after the last injection and transcardially perfused with 300 ml of 0.01M phosphate-buffered saline (PBS, pH 7.35) followed by 500 ml of 4% paraformaldehyde in 0.1 M phosphate buffer (PB, pH 7.35). Lumbar segments of spinal cord (L4 and L5) were quickly removed and post-fixed in the same fixative for overnight and cryoprotected in 0.1M PB buffered 30% sucrose until the segments sank to the bottom. Fixed tissues were processed through graded alcohols and xylenes and paraffin-embedded on the next day. Paraffin-embedded tissue sections of 25 μm thickness were cut using a cryostat and mounted onto slides. Sections were rinsed in 0.01 M PBS for 3 × 10 min. For fluorescence immunostaining of PKCγ and NR1, sections were blocked for 30 min in PBS containing 1% BSA, 5% donkey serum and 0.3% Triton X-100. After rinsing 3 × 10 min, sections were incubated overnight at 4°C with a primary antibody against PKCγ (1:100; mouse anti-rabbit polyclonal; Zymed Laboratories Inc., South San Francisco, CA) or NR1 (1:250, rabbit anti-mouse monoclonal; Novus Biologicals, Littleton, CO). After rinsing in PBS (3 × 10 min), the secondary antibody (1:500; CY3 or FITC conjugated donkey anti-rabbit IgG, Jackson ImmunoResearch, West Grove, PA) was added and sections were incubated for 2 h at room temperature. These sections were again rinsed with PBS and slip-covered. For double staining, the second primary antibody was added after incubation with the first primary antibody following the same procedure. Six nonadjacent sections were randomly selected and analyzed using an Olympus fluorescence microscope, photographed with a digital camera, and processed with Adobe Photoshop 7.0.

### Statistical analysis

SPSS 16.0 (Chicago, IL) was used to conduct statistical analysis . All data are expressed as mean ± SEM. The repetitive behavioral measurements were first tested for normality and then tested by repeated-measures of analysis of variance (Two-way ANOVA). MPAE% of morphine as well as expression levels of PKCγ and NR1 were analyzed by one-way ANOVA. All multiple-comparison analyses were followed by post hoc Bonferroni’s correction. The statistical significance was set at a level of *P* < 0.05.

## Results

A total of twenty-four rats were included in the statistical analysis (n = 6 in each group). There were no differences in baseline weight, mechanical withdrawal threshold and thermal withdrawal latency among the four groups in this study.

### Melatonin attenuated morphine-induced hyperalgesia

**Figure 1:**
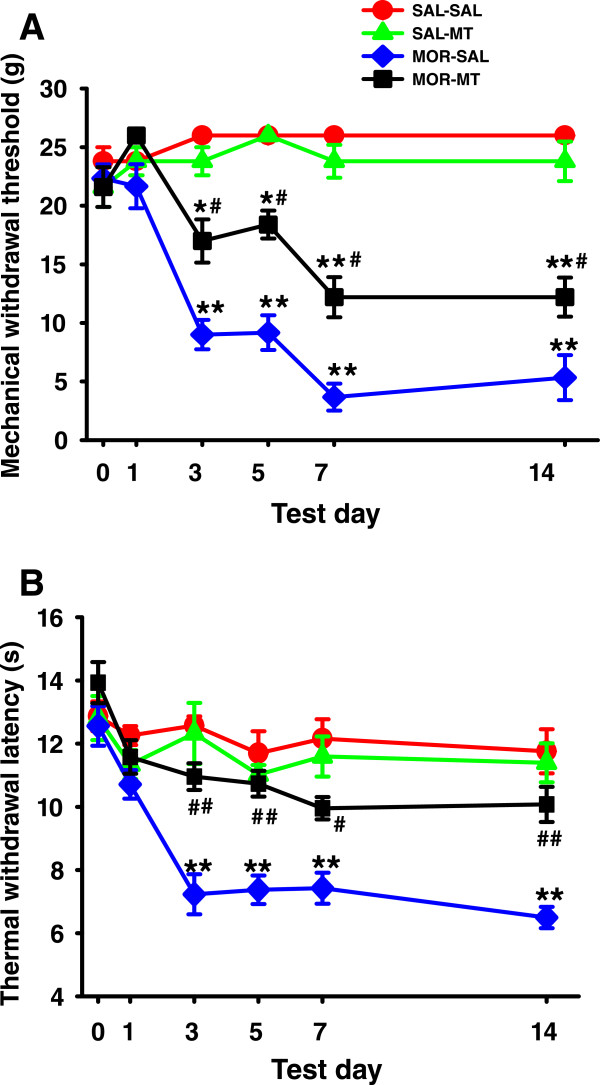
**Melatonin attenuated morphine-induced mechanical and thermal hyperalgesia. A**: Hindpaw withdrawal threshold to mechanical stimulation. **B**: Hindpaw withdrawal latency to thermal stimulation. Mechanical withdrawal threshold and thermal withdrawal latency were both gradually decreased and shortened, respectively, in rats that received morphine (10 mg/kg, s.c.) alone from day 3 to 14. Co-administration of morphine with melatonin(10 mg/kg, i.p.) significantly prevented the decreased withdrawal threshold and latency from day 3 to day 14. Six rats were included in each group. *P < 0.05 and **P < 0.01, compared with SAL-SAL; # P <0.05 and ## P < 0.01, compared with MOR-SAL. Data are presented as mean ± SEM.

Effects of melatonin on mechanical allodynia and thermal hyperalgesia in rats are shown in Figure 1. The hindpaw withdrawal threshold and latency of all rats among four groups with different treatments showed no significant difference on day 0 and 1 (*P* > 0.05). The treatment with morphine (10 mg/kg, s.c.) and saline (MOR-SAL) resulted in a progressive decreased withdrawal threshold to mechanical stimulation and shortened latency to heat stimulation during the post-injective 3–14 days. This is statistically significant compared with those in groups of saline-saline (SAL-SAL) and saline- melatonin (SAL-MT) (*P* < 0.001). Such increased mechanical and thermal sensitivity of animals following repetitive morphine treatment was greatly attenuated by co-administration of morphine with melatonin (10 mg/kg, i.p.) in the group of morphine-melatonin (MOR-MT). The treatment of saline or melatonin alone did not change the sensitivity to the nociceptive stimulation.

### Melatonin reduced morphine tolerance

**Figure 2:**
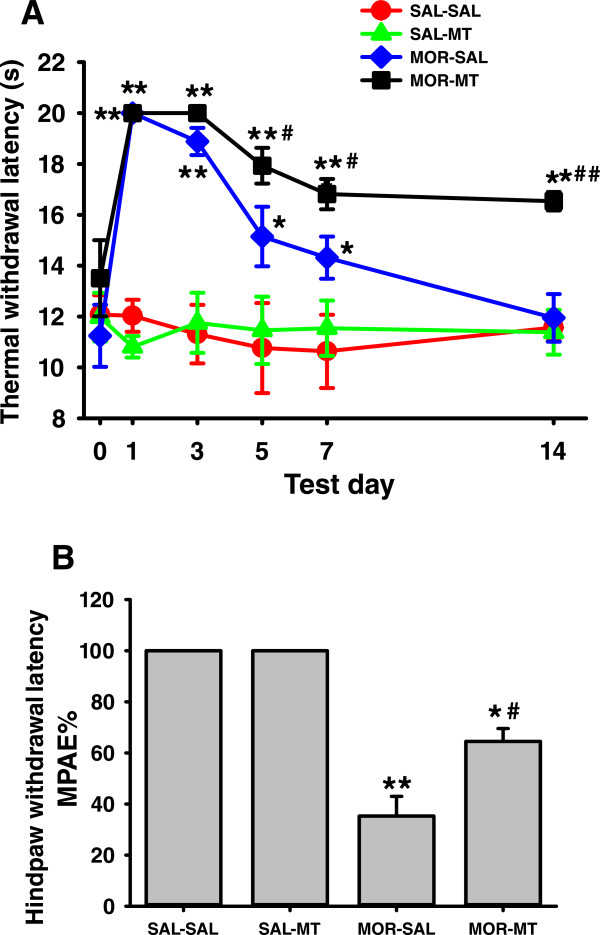
**Effect of melatonin on the morphine tolerance.** The development of the tolerance to morphine’s analgesic effect was assessed by the hindpaw thermal withdrawal latency at 60 min after co-administration (10 mg/kg melatonin and/or 10 mg/kg morphine) from day 1 to 14 **(A)**, as well as MPAE% of morphine at 60 min after administration of morphine (20 mg/kg) alone on day 15 **(B)**. **A**: Injection of morphine (10 mg/kg) significantly increased the thermal withdrawal latency in rats receiving administration of morphine on day 1. However, such an analgesic effect gradually decreased and then disappeared (tolerance) from day 3 to 14 after repeated treatment of morphine. Co-administration of 10 mg/kg melatonin reversed the analgesic effect of morphine. **B**: The MPAE% of morphine (20 mg/kg) significantly decreased in rats receiving repeated administration of morphine on day 15. However, the decreased MPAE% owing to morphine tolerance was reversed by consecutive 14 days co-administration of morphine with melatonin. MPAE% = [(test latency - basal latency)/(20–basal latency) × 100% (20 s as the cut-off time). *P < 0.05 and **P < 0.01, as compared with the SAL-SAL group at the same time point; # P <0.05 and ## P < 0.01, as compared with the MOR-SAL group at the same time point. Data are presented as mean ± SD for 6 rats per group.

Morphine tolerance and effects of melatonin on morphine tolerance in inhibiting thermal hyperalgesia are shown in Figure 2. Morphine (10 mg/kg, s.c.)-induced analgesic effect on the thermal hypersensitivity was quickly decreased 5–7 days after repetitive morphine treatment. Melatonin (10 mg/kg) treatment successfully rescued the analgesic effect of morphine and such action lasted for at least two weeks. Melatonin (10 mg/kg) treatment at this dose alone did not significantly affect the thermal hypersensitivity (Figure 2A). In Figure 2B, The MPAE% of morphine decreased about 60% in MOR-SAL group, compared to the SAL-SAL group (*P* < 0.001), on day 15. The decreased MPAE% owing to morphine tolerance was reversed by consecutive 14 days co-administration of morphine with melatonin. The MPAE% in MOR-MT group was only decreased by about 30% compared with SAL-SAL group, suggesting MPAE% was significantly improved in rats of the MOR-MT group (*P* = 0.001). MPAE% between groups of SAL-MT and SAL-SAL was not significantly different (*P* > 0.05).

### Melatonin inhibited the morphine-induced increased expression of PKCγ and NR1 in the spinal cord

**Figure 3:**
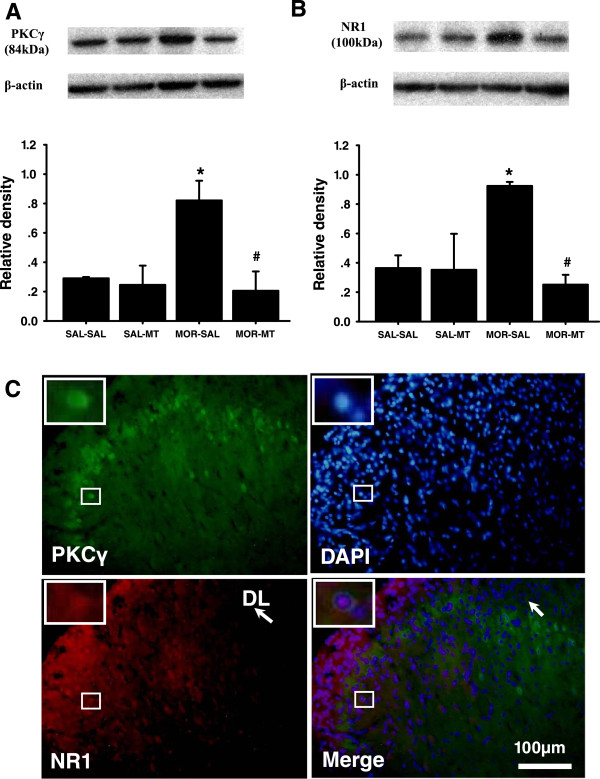
**Effects of melatonin on morphine-induced increased expression of PKCγ** **and NR1 in the spinal cord.** Western blot shows expression of PKCγ **(A)** and NR1 **(B)** in the spinal cord dorsal horn (n = 3) in each sample following treatment of morphine with or without melatonin for consecutive 14 days. Data are presented as mean ± SEM. *P < 0.05, compared with SAL-SAL group # P < 0.05, compared with MOR-SAL group. **C**: Co-localization of spinal PKCγ and NR1. There was co-localization of PKCγ and NR1 immunoreactivity in the superficial layers (I and II) of the spinal cord dorsal horns at the lumbar (L4) level. Spinal cord samples were taken from rats receiving a combination of morphine and melatonin for consecutive 14 days (n = 3). Blue: DAPI for nucleus. Scale bar: 100 μm. DL: the dorsolateral part of the spinal cord dorsal horn.

Western blot analysis showed that, following treatments of morphine and/or melatonin for consecutive 14 days, expressions of PKCγ and NR1 proteins were greatly increased in MOR-SAL gourp, compared to the SAL-SAL group(*P* > 0.05). The increased expression of PKCγ and NR1 was significantly reduced by co-administration of melatonin with morphine in the MOR-MT group, as compared to that in the MOR-SAL group (*P* = 0.038; *P* = 0.025) (Figure 3A and B). Melatonin treatment alone caused alterations of expression of neither PKCγ nor NR1. A co-localization of PKCγ and NR1 could be detected by immunohistochemistry in superficial layers (I and II) of the spinal cord dorsal horn in rats treated with a combined morphine and melatonin for 14 consecutive days.

## Discussion

The present study showed that morphine-induced hyperalgesia can be attenuated by co-administration of morphine with melatonin over a 2-week period. Moreover, co-administration with melatonin also prevents a 2-week morphine treatment regimen-produced morphine analgesic tolerance. The increased expression of PKCγ and NR1 in the spinal cord of morphine exposed rats is also inhibited by co-administration of morphine with melatonin. These results indicate that melatonin treatment can successfully alleviate morphine-induced hyperalgesia and tolerance probably through inhibition of PKCγ and NR1 activities in the spinal cord.

Morphine-induced hyperalgesia and tolerance are two different phenomenons. Morphine-induced hyperalgesia is a paradoxical increase in pain sensitivity that develops after short- and/or long-term morphine exposure [2], while morphine tolerance is a phenomenon in which repeated exposure to morphine results in decreased analgesic effect of the drug or a need for a higher dose to maintain the same analgesic effect, reflected in a rightward shift of the dose–response curve [2]. However, morphine-induced hyperalgesia and tolerance exhibit similar clinical manifestations, e.g., to reach an adequate analgesic effect, it is necessary to increase doses of morphine over time [3]. In our study, nociceptive thresholds and MPAE% of morphine in rats were progressively decreased by administration of morphine over a 2-week period, demonstrating the development of morphine-induced hyperalgesia and morphine tolerance.

Melatonin is a potent neuromodulator, and plays an important role in physiologic and neuroendocrine functions’ regulation via the high-affinity MT_1_ and MT_2_ receptors [23, 29]. Studies have suggested an interaction between melatonin and opioids, i.e., melatonin can not only enhance the analgesic effect of opioids, but also reverse morphine-induced tolerance and dependence [23, 26, 30]. Our study further confirmed these observations. In regards to morphine associated hyperalgesia, melatonin can ameliorate the descending basic nociceptive threshold in rats that received chronic treatment of morphine, indicating that melatonin can prevent the development of morphine-induced hyperalgesia. In regards to the tolerance to morphine’s analgesic effect, melatonin can attenuate the decreasing MPAE% of morphine in rats that received chronic treatment of morphine, suggesting melatonin can prevent the development of morphine tolerance. it is noteworthy that, although in this study melatonin attenuated morphine tolerance when daily co-administered with morphine for 14 days, the morphine analgesic effect was not affected in those rats exposed only to melatonin when a single dose of morphine was administrated only on day 15, suggesting that repetitive, co-administration of melatonin with morphine maybe necessary to demonstrate the impact of melatonin on morphine anti-nociception. This observation is consistent with the previous reports [23, 30, 31].

Morphine-induced hyperalgesia and tolerance are complex physiopathological conditions involving adaptations at multiple levels in both CNS and in peripheral tissues. Intracellular second messenger systems such as PKC have been shown to modulate NMDA receptor activation and play a critical role in molecular mechanisms of morphine-induced hyperalgesia and morphine tolerance [5, 10, 15]. A series of studies suggest that μ-opioid receptor activation induced by morphine treatment may initiate G protein-mediated PKC translocation and activation, cause a removal of the Mg^2+^ blockade from the NMDA receptor that allows for an increased influx of Ca^2+^ [5]. Activation of PKC can modulate μ-opioid receptors responsiveness, resulting in desensitization of the μ-opioid receptors [12]. Consistent with our previous observation [15], in the present experiment, we have demonstrated that expression of PKCγ and NR1, which are co-localized within superficialI&II layers of the spinal cord dorsal horn, were greatly increased in the dorsal horn of rats that were exposed to morphine. We further demonstrated that such increased expression of PKCγ and NR1 was significantly inhibited by co-administration of melatonin with morphine. These findings may support the idea that melatonin-induced alleviation of morphine-induced hyperalgesia and tolerance is probably mediated through inhibition of the activity of PKC/NMDA pathway. Besides, other possibilities such as activation of opioid system and benzodiazepine-GABAergic pathway may be considered as well [25, 26].

Taking together, our study support the possible mechanism underlying the effect of melatonin on morphine-induced hyperalgesia and tolerance is probably through inhibiting the prolonged activation of μ-opioid receptor-induced PKCγ activity and the increased activity of NR1, thus decreasing the influx of Ca^2+^, reducing the increased neural excitability and gliocytes activity at the spinal cord level. However, our current study does not clarify how melatonin inhibits the PKC/NMDA pathway. A possible mechanism is that melatonin decreases intracellular cyclic adenosine monophosphate (cAMP) level by inhibiting the activity of adenylate cyclase [3]. Data we have provided in this study is limited, further studies on regulation of PKC and NMDA receptors in melatonin’s modulation of morphine-induced hyperalgesia and tolerance are urgently needed.

## Conclusions

Our study suggests an idea that co-administration of melatonin with morphine may be a helpful strategy for enhancing the clinical use of morphine in treating chronic pain and reducing the hyperalgesia and tolerance following repetitive morphine treatment .

## Abbreviations

PKC: Protein kinase C
PKCγ: Protein kinase C gamma
NMDA: N-methyl-D-aspartate
NR1: NMDA receptor 1
MPAE%: Percent of maximal possible anti-nociceptive effect.
